# How do people with long‐term mental health problems negotiate relationships with network members at times of crisis?

**DOI:** 10.1111/hex.12620

**Published:** 2017-10-10

**Authors:** Sandra Walker, Anne Kennedy, Ivaylo Vassilev, Anne Rogers

**Affiliations:** ^1^ NIHR CLAHRC Wessex Faculty of Health Sciences University of Southampton Southampton UK

**Keywords:** isolation, mental Health, peer support, relationships, social networks

## Abstract

**Background:**

Social network processes impact on the genesis and management of mental health problems. There is currently less understanding of the way people negotiate networked relationships in times of crisis compared to how they manage at other times.

**Objective:**

This paper explores the patterns and nature of personal network involvement at times of crises and how these may differ from day‐to‐day networks of recovery and maintenance.

**Method:**

Semi‐structured interviews with 25 participants with a diagnosis of long‐term mental health (MH) problems drawn from recovery settings in the south of England. Interviews centred on personal network mapping of members and resources providing support. The mapping interviews explored the work of network members and changes in times of crisis. Interviews were recorded, transcribed and analysed using a framework analysis.

**Results:**

Three key themes were identified: the fluidity of network relationality between crisis and recovery; isolation as a means of crises management; leaning towards peer support. Personal network input retreated at times of crisis often as result of “ejection” from the network by participants who used self‐isolation as a personal management strategy in an attempt to deal with crises. Peer support is considered useful during a crisis, whilst the role of services was viewed with some ambiguity.

**Conclusions:**

Social networks membership, and type and depth of involvement, is subject to change between times of crisis and everyday support. This has implications for managing mental health in terms of engaging with network support differently in times of crises versus recovery and everyday living.

## 1 INTRODUCTION

Social interventions for mental health recovery and management have increasingly been viewed as an alternative to more traditional means of management especially in relation to those from underserved communities.^1^ Clinical treatment models of social interventions,^2^ forming the bases of a purported biopsychosocial model have seemingly failed to prioritize the “social” elements; however, interventions which prioritize participation have suggested positive outcomes associated with asset‐based approaches, trusting relationships and resource‐seeking to enhance community participation.^3^ Social participation interventions, such as social prescribing or community referral which have been seen as a means of improving access to psychological treatments and other resources which support mental health, bring to the fore the need to illuminate the role, properties and function of network mechanisms.^4^ A network perspective offers opportunities to explore the ways in which the quality of social relationships may impact on mental health^5^ and to address the latent assets and resources which may be available to people in need of condition management and support which lie outside the formal health‐care delivery system.^6^ Support from social networks has been shown to make a contribution to improved health outcomes for people with long‐term conditions and in the genesis of mental health (MH) problems and utilization of services.^7^, ^8^ The utility of social network resources depends on successful activation of connections that can provide access to relevant information or support.^9^

Exploring the role and function of social ties is relevant for understanding the support and resources that are leveraged in the trajectories of those experiencing MH problems in everyday life recovery and in times of crises. Previous research suggests that one response to crisis is to identify those most able to provide support from a larger group (selective activation) with the consequence that those able to secure “adequate” network resources seemingly report better outcomes than those who “injudiciously” select network ties.^7^ A “social safety net,” comprising community organizations and health‐related network ties has the potential to reduce the utilization of MH services^10^ by providing emotional and direct alternative support for self‐management activity. People, animals and material objects are key to providing support and linking people to needed resources in a person's personal network. For example, online peer support has been found to provide benefits through social connectedness, feelings of group belonging and the sharing of strategies and narrative for coping with the challenges of living life with a MH problem.^11^ Diverse networks (including strong and weak ties) may be better placed (compared to more restricted network types) to support long‐term condition management, because of the availability of increased opportunities for negotiating relationships with network members and resources.^6^ Connections to and interaction with objects, places, pets and activities are also likely to be relevant to understanding the crises and recovery “work” of those with a MH problem.^12^ However, relatively little is understood about the way in which network members provide support and resources for management, and the difference in how people negotiate network support requirements at different times (eg, in times of crisis versus recovery). Here, we utilize network mapping as an heuristic device to explore people's personal networks to examine the support available to manage MH and explore relationships with different network members at times of crisis.

Crisis represents a negative event in life. However, it can also be an opportunity for growth.^13^ For the purposes of this study, crisis is defined as the point at which mental distress becomes overwhelming or unmanageable to the extent that the experience of it disrupts everyday life. It is a multifaceted process which can be understood as a trajectory that can be recognizable but is not necessarily linear.^14^

## 2 METHOD

The project forms part of a larger programme of research carried out through Wessex CLAHRC exploring self‐directed support, people's social networks and links to local community resources in the management of LTC. At the beginning of the study, a Patient and Public Involvement event was held at a local recovery college, and people with MH problems helped co‐produce the project and its design. The main changes that occurred as a result of this consultation were that networks in times of crisis were to be considered alongside day‐to‐day networks. SW carried out semi‐structured interviews and mapping with participants in a community‐based context on the South Coast Hampshire. SW then independently coded the data whilst regularly discussing the emerging themes with AR, IV and AK who also independently coded six interviews to enhance rigour. The whole team met regularly to discuss on‐going analysis and to discuss, explore and confirm emergent codes.

Twenty‐five participants were recruited, of which nineteen were female and six male.^1^ All participants were white British; ten were married with the remainder single, eight lived alone; ten were unemployed, nine in occupation, including voluntary (n = 1), three were retired and three considered themselves disabled; eleven considered themselves to live in an affluent area with the remainder less so with three stating their area as deprived. For further demographic information, see Table 1.

**Table 1:** Demographics

Participant No	Gender	Age	Employment status	Civil status	Living alone?
1	Female	39	Disabled	Single	No
2	Female	57	Voluntary work	Divorced/Separated	Yes
3	Male	41	Part‐time	Divorced/Separated	No
4	Female	56	Unemployed	Single	Yes
5	Female	29	Full‐time	Single	Yes
6	Male	42	Part‐time	Married	No
7	Male	56	Retired	Married	No
8	Female	39	Unemployed	Married	No
9	Female	39	Unemployed	Married	No
10	Female	56	Part‐time	Divorced/Separated	Yes
11	Female	49	Full‐time	Married	No
12	Female	47	Part‐time	Married	No
13	Female	62	Retired	Married	No
14	Male	51	Unemployed	Single	Yes
15	Female	55	Part‐time	Married	No
16	Female	31	Part‐time	Divorced/Separated	Yes
17	Female	34	Unemployed	Single	No
18	Female	40	Unemployed	Married	No
19	Female	35	Unemployed	Single	Unknown
20	Female	52	Disabled	Single	No
21	Female	32	Disabled	Single	No
22	Female	47	Unemployed	Married	No
23	Male	57	Retired	Divorced/Separated	Yes
24	Female	53	Unemployed	Divorced/Separated	Yes
25	Male	42	Unemployed	Single	No

### 2.1 The sample

Participants in this study were considered eligible if they were aged over 18 and had been diagnosed with a MH problem by either secondary care services (22) or their GP (3). Participants were recruited via venues which included a local recovery college and community‐based non‐statutory MH support groups. As these groups already involve a degree of self‐management, it is possible that participants may represent a more motivated group of people with MH problems.

Potential participants who responded to adverts of the study placed in the aforementioned venues were recruited to the study. A consent form was signed once the participant was conversant with the study. Convenience sampling was used and those that came forward for selection, if they met the inclusion criteria, were included regardless of gender or demographic status. There was a purposive element to this, however, as a formal diagnosis of a MH problem as an adult was a pre‐requisite for inclusion in the study.

### 2.2 Data collection

Face‐to‐face semi‐structured interviews were carried out, by the lead author, between October 2015 and April 2016 at a location convenient to the participant. Participants were asked to map social networks using a concentric circle diagram which was adapted from previous studies.^15^ Participants placed network members on the map. They were asked to consider the inner circle as most essential to their MH, the middle circle as very important and the outer circle as less important than the other two circles. Network members could be people, places, activities or objects, in fact anything that the person considered to be valuable to them in terms of managing their MH. No maximum number of network members was prescribed, and participants were able to list as few or as many as they considered relevant to their situation.

In addition to their day‐to‐day maps, participants were asked to indicate how the map would change in a crisis. Four participants created another map to indicate crisis networks, but the majority of participants used different colour pens to circle or underline the network members that would remain in the network in crisis and arrows to indicate where network members might move between circles of importance. Interviews lasted between 25 and 100 minutes and explored the role and key attributes of individual network members to MH management on a day‐to‐day basis and during crisis, thus detailed information was collected about the contributions each member made to MH management at these times.

Ethical approval was obtained from the University of Southampton research governance office.

### 2.3 Data analysis

We took an inductive approach for the analysis. Data comprised of audio recordings and verbatim transcripts of 25 semi‐structured interviews; field notes were taken at the time of interviews and the network maps were completed at the time by each participant. Data were categorized firstly by participant, interrogated to address the title question then coded accordingly. Immersion in the data was achieved by repeated listening to the recordings by the lead author, then the transcript and associated notes were further explored and each coded. Emergent categories were checked and discussed in data analysis clinic with the other authors, and six of the interviews were analysed by the whole research team. The themes were then compared and emerging codes revisited across the data as part of a process of reflexive dialogue.

## 3 RESULTS

Here, we present data and emergent themes most pertinent to management of crisis.

### 3.1 The fluidity of network relationality between crisis and recovery

With some people operating in crisis mode for much of the time, participants described crisis as a fluid experience, which from recognizing triggers from past experience had to be managed in the moment. Participants suggested that the direction of travel in this situation is not always the same, some found that crisis could be averted by certain activities or interactions and others found that reaching out for help and not getting it could precipitate crisis unexpectedly.

All participants reported reduced networks in times of crisis (see Table 2). Those members most often leaving (sometimes temporarily) or moving to the periphery of a personal network in crisis were members representing a source for social involvement (eg, engagement with a voluntary organization).

**Table 2:** Network numbers

Part. no.	Dav‐to‐d ay	Crisis	Part. No.	Dav‐to‐d av	Crisis
1	18	15	13	21	3
2	33	9	14	14	3
3	21	12	15	22	4
4	15	3	16	26	11
5	15	4	17	35	9
6	16	5	18	15	7
7	20	5	19	10	4
B	19	3	20	20	B
9	20	6	21	20	5
10	29	5	22	21	2
11	26	9	23	18	6
12	16	3	24	21	4
			25	10	2

Members most likely to remain in contact during crisis, tended to be spouses or very close family members. They were described by the participants as those who were reliable whatever the circumstances and were able to accept them despite their MH problems and allow the participant to “be themselves.”

we'll just sit with it and it will be OK. It's not always quite like that occasionally it will create a bit of stress between us but I think the fact that we can really communicate. He's the first partner I've had where I've been able to really feel I can communicate and they get it. My previous partner just didn't get it, my ups, my downs, and my nuttiness. P8

I'll just splatter things out and then I'll just mumble it away and I'll just come out with all sorts of madness but my wife will know and she'll sit and we'll pick through it and she'll help me. P6

The possession of a crisis management plan is part of modern day MH care and reflecting the anticipation of crises in the future, some of the participants who had previously experienced a MH crisis, managed network members in such a way as to help maximize the chances of being prepared for another crisis should it occur. One such measure was taking steps to protect the most valued relationships from the consequences of being in crises. This included limiting the amount of information shared with others due to the fear of overburdening them with support expectations, being aware of the responsibilities that they already carry and concern for their wellbeing:

a very close friend, his wife has got cancer at the moment so I find I have to be careful because I could overload him with stuff, he's got his own issues. P23

In periods of non‐crisis, whilst managing intense emotions seemed to be a daily endeavour many participants described spreading the load by getting support from a variety of different network members. Participants with a diverse day‐to‐day network seemed more geared towards this end than those that did not. This diversification “strategy” seemingly acted to prevent individuals from becoming overburdened should they be exposed to the work of supporting someone in crisis. For one participant, close relationships were so valuable to her that she actively avoided them in crisis tending to prefer utilizing services so that she could still interact with her family members when she was well:

The only people I haven't let know is my brothers and also my Mum's partner because I don't want them to think oh my goodness they're going to have to help me with this, that and the other. I want to be able to still have that break to see them without having to discuss the whole situation with every single person that I know. P20

The value of this relationship cannot be described as strategic, as a resource, but rather as carrying intrinsic value through sense of self and concern for others, which is contrasted with the strategic use of services. As such, this cannot be seen as rationing support.

Relationships were not always viewed so positively, and some were seen as more precarious. Participants indicated that the emotional work required in close relationships was sometimes too hard to cope with:

if mum answers the phone she will sense in my voice and she will get fairly upset. It doesn't seem like it's in sympathy it seems like she's angry with me for making her feel sad, I'm sure she is concerned really but it doesn't come over like that. So I will avoid, I will go round it might be quite briefly at a good time in the day maybe but sometimes I might not see them for three or four days. P23

Parents featured significantly here, as did children, who were seen as joyful and draining.

I just happened to mention it to my parents, the reaction I had was massive, it was a typical sort of parent/child syndrome and I just thought, I listened to it all and it had been something that's repeated time and time again and I thought for the first time that's your problem not mine but it did hurt, it still really hurt so I thought for all the good I thought I was doing they just wiped it all out in one foul swoop and they can do that. P24

Here, a participant provides an example of the negotiation of relationships with her husband and her mother, involving positive and negative elements:

It can be difficult at times because there is a triangle with her and my husband and I which doesn't always sit well with either of them but we've muddled through and generally it's OK but there can be times when it's quite strained and then I feel like I'm piggy in the middle and that doesn't help but when I'm having a really bad time with mental health. P22

**Figure 1:**
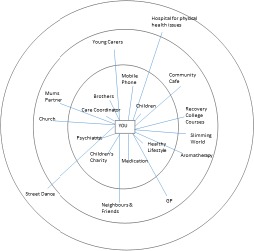
Day‐to‐day example Network

**Figure 2:**
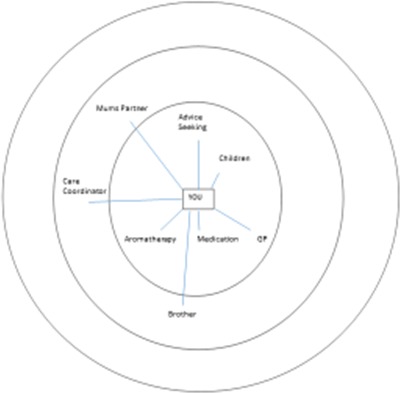
Crisis example network (same participant)

The changing nature of networks from day‐to‐day to crisis is presented in Figures 1 and 2.

Figure 1 represents a participant who was a single mother with four children. It shows a diverse network representing her day‐to‐day network, incorporating family, friends, services both voluntary and statutory and activities.

By contrast, the diagram (Figure 2) illuminating the network of the same participant in crisis is significantly smaller. Notably, all of the activities in the outer circle have “disappeared” along with many of those in the middle circle. The participant's partner, medication and her children are the only network members that remain in place. The care coordinator remains on the map but is relegated to the middle circle and her psychiatrist is removed. Aromatherapy moves up in importance as does the input from the GP. All social activities are gone and only one brother remains, a situation she stated was due to his having had MH issues too so he was able to understand how she felt. Advice seeking is added to the map and described as something undertaken predominantly when in crisis to provide reassurance from multiple points at this time.

Whilst the second map is reduced in scale, it is still relatively diverse. This was the case for 16 participants and may indicate that diversity of networks as best for illness management might still hold in a crisis situation, although in a more condensed form.

### 3.2 Isolation as a means of crises management

Isolation appeared as a characterizing feature of managing crisis. Isolationism was considered to have both a positive and a negative function but was primarily viewed as a strategy for managing crisis. The role of isolation varied between individuals. For some, the sense of needing to “*hunker down” P2* for safety: *‘Get back in the womb almost and just stay there*.’ *P8*. The peace and quiet available in this space acted as a time for healing, for reflection, to allow private expression of emotion and a time to regather strength.

I feel safe, if there is no one there then obviously, if there's no one around to bother you then you're fine, no one there to bother you so you're safe and that's just the way I Iike to be, safe, after feeling very much alone for so long it's just nice to be quiet and peaceful. P25

I find there are times when I need to have that quiet time, that time on my own, that time to be still, a time to not see anybody so that I can work through or have a hissy fit or cry it out and then you are ready to talk. P2

For some, it clearly provided an escape from the demands of others at a time when they were less able to function socially:

I can find that quite a drain because when I feel low I just don't want to interact with anybody and I have to really force myself. P18

Isolation allowed time to watch and wait and thus served a potentially protective role in seeing whether this was a crisis or just a phase that would pass. Additionally, isolation acted to maintain dignity at a time where the individual has noted they are becoming unwell ‘*then I just go quietly nuts’ P8*; and needing to limit stimulation *‘rest, relax and let things tick over’ P12*. There was a sense from participants that no one can help until the time is right. Although this isolation or withdrawal is an essential part of the process, it can also become part of the problem if it goes on too long.

I don't think it does help, I think I falsely think that I can sort it out on my own and it will pass but what tends to happen is the days pass but the feelings don't pass so I actually make myself worse by isolating myself. I think that I can get myself out of it…… I think sometimes I can but I just need a couple of days of panic. P24

### 3.3 Leaning towards peer support in times of crises

The foregrounding of the relevance of peer support, where peers represent someone with lived experience of MH problems rather than formal peer workers housed with services, was a feature of crisis network configuration. Peer support was noted in 18 participant day‐to‐day networks and 14 crisis networks. In two instances, the peers' position within the network remains unchanged; in four cases, they decrease in importance; and in two cases, they increase in importance during crisis (see Table 3). Eleven networks had peers positioned within the inner circle and of these, 8 remained there during crisis suggesting that peer support remains useful throughout crisis in a way services did not appear to.

**Table 3:**
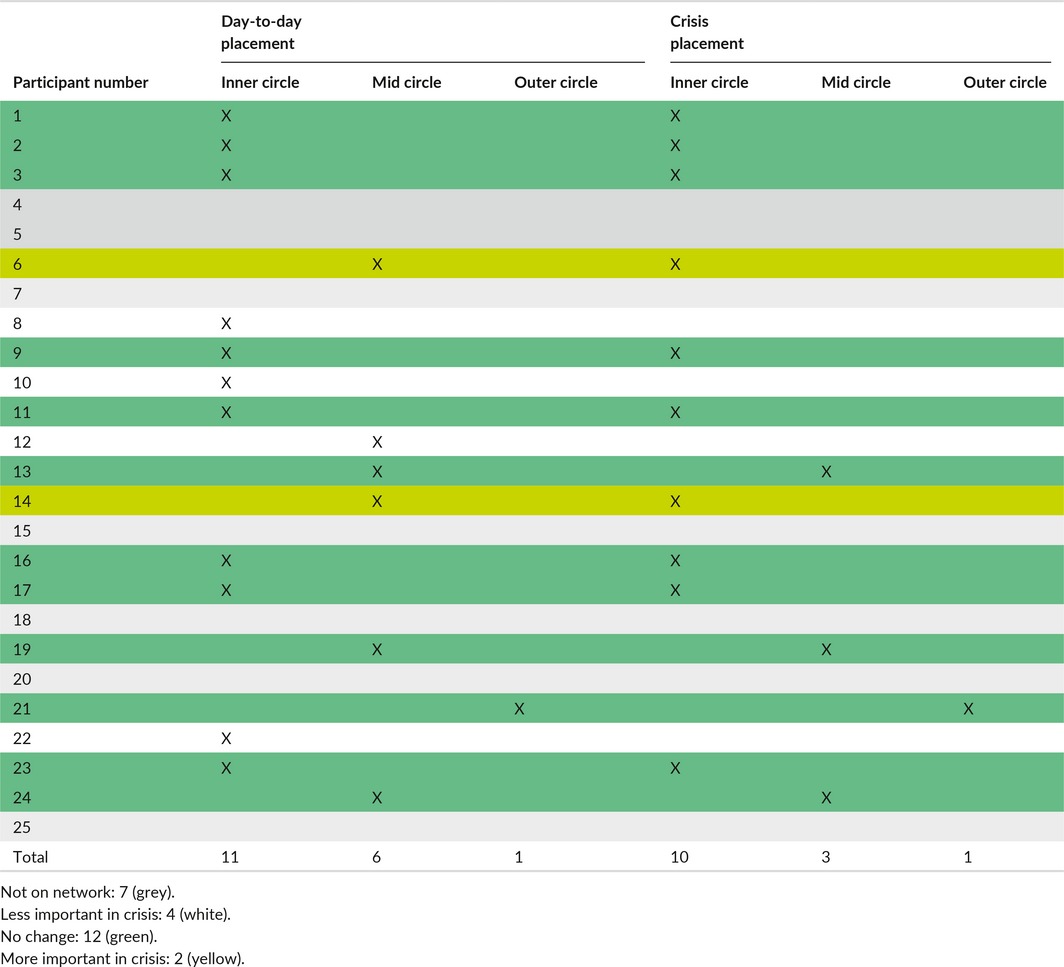
Peer member network positioning

Peer network members appear to provide a basis for rootedness and empathy which is meaningful, and emotional support is seen to be useful and sensitively delivered:

if I was in a place where something was really wrong and I couldn't work it out I would go there at the drop of a hat just go and…. see the peers that I used to go to group with. I'm still in contact with some of them. Peers would be there. Also they're quite important but if I was in crisis I would put them there [indicates moving to inner circle]. P6

Many participants contacted peers online and found that they could continue this level of communication further into crisis then actual face‐to‐face interaction:

and I think my internet friends I've probably got more in common with just because it's not accident of circumstance, well with a lot of them it is accident of circumstance but we've had to actively choose to be together and obviously because when we're talking on line it's easier to share articles and bits of information and stuff that we're doing we're more likely to talk about ideas. P21

This peer support was relevant in terms of acceptance for who one was and how one might behave. It can also function as a means of responsive containment as illustrated by one participant when he says:

I used to be wrapped up in a lot of football violence when I was younger and things like that, so that was my only positive way of getting that aggression out would be to play football in a safe way because the manager and everyone knew my condition so if I was getting a bit too aggressive they'd pull me on it and say listen but it was all done in a positive way and for me that was always my way of releasing that positively. P6

By contrast, mental health services seemed to elicit more equivocal endorsement in terms of the support provided. Although twenty‐two of twenty‐five participants were open to secondary MH services at the time of interview, only sixteen of day‐to‐day networks featured MH services as a member and this reduced to twelve in crisis. Of this sixteen, when going into crisis, five participants maintained the importance of services in their circles; five found they increased in importance; six found they reduced in importance; four used services day‐to‐day then dropped them in crisis; and one used services in crisis only. So, in ten of the sixteen cases, services maintained their position or increased in importance during crisis (Table 4).

**Table 4:**
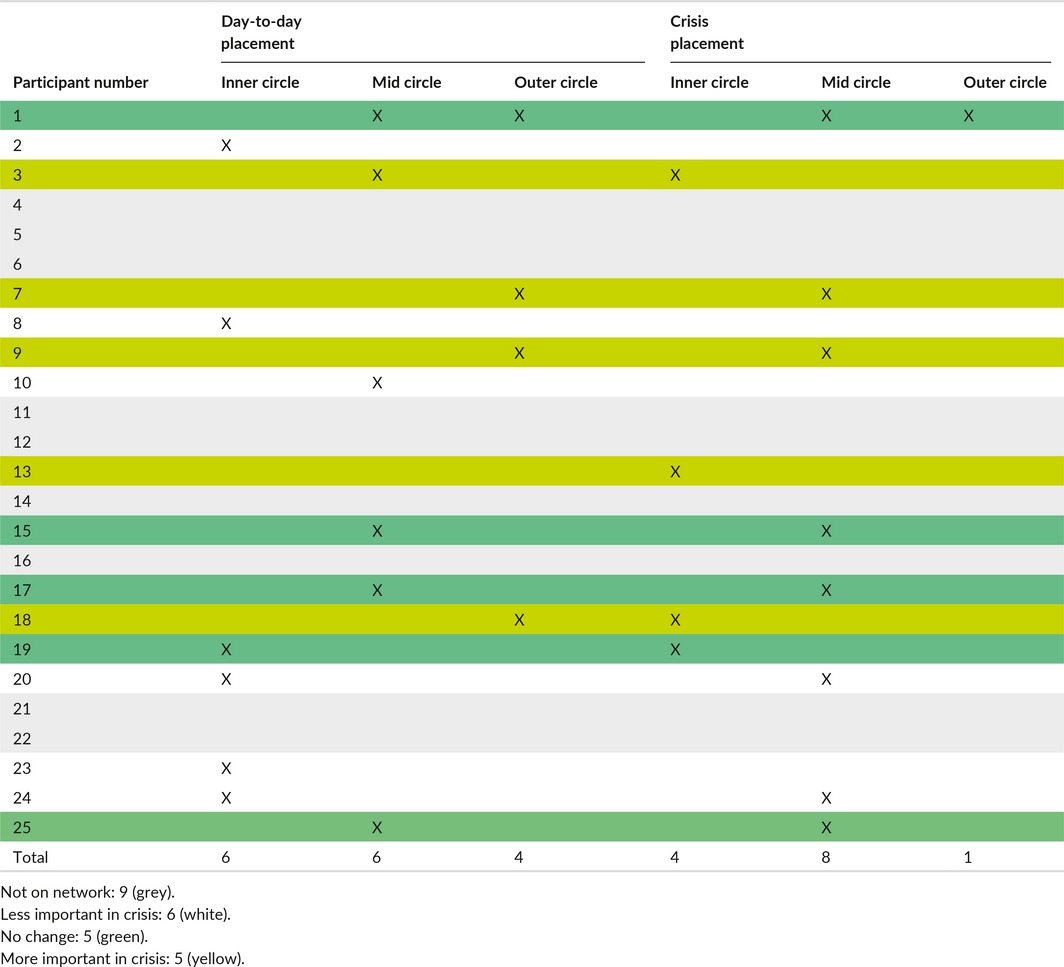
Mental health service network positioning

Although not always present in the network, all but one participant mentioned services during their interview. Twenty cited systems' issues as problematic. One participant illustrates this when she describes what happened after she approached the service in an effort to prevent crisis:

After I left there I had a letter a few weeks later, honestly the amount of paper that was in there I couldn't believe it and I cried not for myself but for the even one individual because it said if we do not hear from you within 18 days we will assume that you do not need, no longer need our assistance. To me that was just disgusting. P5

However, despite its flaws, many participants would rather there was a service than no help at all:

I have [MH Nurse], which has got some good points and some not so good points. At times I think she's a sign‐poster I understand that but I think she could possibly do more than she actually does but I'm grateful to have her there. P24

I think if I got really unwell I think the Crisis team would be in there even though they weren't very helpful. P18

Whilst the stated role of many MH services is crisis support, the participants in this study predominantly found them useful in crisis prevention, in an effort to ward off the crisis, or when emerging from crisis when they required help reconnecting with life again and making sense of what had happened. The service may also have an important role in supplementing self‐isolation and strong tie support in crisis.

I suppose when you are quite low like me at times and in a crisis quite a bit of the time it feels like you want an instant fix, you don't want to spend three months with a well I suppose you might get some friends out of this or I suppose it could work, so it's almost like I'm almost trying to feel better but I don't want to do the work and I suppose that's because my motivation is not brilliant all the time. P23

Here, indicating that he had been encouraged to socialize more whilst in crisis which was unhelpful at the time of crisis but may well have been helpful to ward off the crisis or help him reconnect to others on emerging from crisis. The use of services could reduce the burden on strong ties, thus despite them not being ideal they remain important, as illustrated here:

When I'm not well [uses services] so as and when needed. These people that support me but I don't really like them…. A necessary evil…. They're necessary, I have to do it. It's like medicine but I hate it, every minute of it. P9

It also appears that the skills that participants learned during their contact with services pre‐ and post‐crisis were most useful in terms of everyday coping and prevention of further crises:

Well I practice mindfulness. I try to do that with any stress or situation because I hear voices and sometimes it can be really stressful so I try mindfulness activities and things around that like mindfulness of washing up or I quite like crystals so I'll sit and do stuff with my crystals mindfully and things. P8

The relationships that are built with individual practitioners within services were also cited as useful by 14 participants.

so I guess the crisis team would be [on the network map], but it was great that they came out to me every day but I would certainly hold the intimacy that I have with [CPN] being there care wise a lot more than crisis. P17

## 4 DISCUSSION

Social networks provide the structural elements of mechanisms for social interactions through which personal social systems of support are built, and the means through which individuals act and react to network members in their immediate environment. This study has examined the personal social networks implicated in day‐to‐day support compared to crisis management of those experiencing a mental health problem. The configuration of networks provided through the mapping exercise illuminated the dynamic nature of a personal network for those with a mental health problem. Our analysis illuminated how compared to day‐to‐day management, networks change in times of crisis.

Day‐to‐day networks differed from crisis networks in so far as they tended towards greater diversity (ie, containing a mixture of people, activities, pets and places) and thus capacity for social involvement. This can enhance self‐efficacy, the capacity to self‐manage and the leveraging of resources from other people, voluntary and community organizations.^16^, ^17^

In times of crisis, interactions with networks members were more selective tend to shrink, with the participants' spouses or friends most likely to remain in the network. Being in touch with fewer but closer social network members was conceptualized by respondents as a means of ensuring the security of continuing acceptance despite the difficulties presented by a crisis. Interaction with fewer close network members might represent the greater ease of managing the permutations of dishonour, shame, enacted and felt stigma which accompany the onset of a crisis.^18^, ^19^, ^20^

The apparent reduction in the size of the network also seemed to present the bases of managing crises through isolation—allowing withdrawal and the means to cope on one's own. These results of differences in social network member composition between times of crises and day‐to‐day management align with studies that indicate that, notwithstanding the advantages of diverse networks, individuals have to deal with a considerable amount of relational work involved in negotiating a multiplicity of roles and responsibilities across the network. The latter is likely to become more burdensome in crises adding to emotional overload^21^ at a time of extreme emotional turmoil. However, a smaller number of ties at times of crises might reflect the flexibility and adaptability of a personal network. Recourse to a select few in times of extreme distress may indicate a modicum of success of prior investment in the negotiation, navigation and relational work previously undertaken in the building of intimate relationships. In this respect, some weak ties may remain valuable in crisis. Many of the networks in this study still remained diverse, although backgrounded, in an attempt perhaps to protect valued relationships. Whilst this isolating tendency raises the question as to whether over time people who experience a greater number of crises are more vulnerable to the permanent loss of diverse networks and the advantages they convey, changing networks appear to influence management at different time points. Most of the participants had been in crisis before and the day‐to‐day network appeared to represent efforts to prepare for the possibility of future crisis. Isolation appears as a feature of personal crises and network management, and there is an inclination for preferencing the input of peer and friendship support and the questioning of the utility of formal services during crisis itself. The ambivalence about their value may reflect the observation, previous research of the perceived surveillance and punitive restrictions of compulsion included under the mental health act which are associated with the ministrations of formal services.^22^

The results implicate the need to consider strategies for further understanding and promoting sustainable flexibility within networks, to preserve the diverse nature of day‐to‐day social membership. The presence of a range of network configurations implies a rich source of personal social and emotional relationships from which to draw upon in the everyday lives of people with MH problems^23^ and a smaller, closer set at times of crises. Identifying points at which relational work might enhance people's sources of support and access to valued activities is likely to enhance people's quality of life in living with a MH problem.^24^ The latter may provide the bases for on‐going support and a means of preventing crises through social involvement. Diverse networks are more durable over time and avoid the intense intimacy of friends and family so may be experienced as less stressful.^25^ They may provide possibilities to spread the load of support over a large group of people and consistency of relationships which can be returned to once the individual is out of crisis and able to interact again. In this respect, our findings align with studies where the importance of retreating to “safe havens” has been noted^26^, ^27^ and in crisis, some respond by managing alone. Isolation led to mixed outcomes for participants in one study,^27^ for some the crisis resolved and for others it exacerbated the situation a position echoed by participants in this study. So, whilst this pragmatic coping technique “gets the job done”,^28^ it has associated costs which then may need addressing.

The finding that isolation and a shrinking network can confer benefits at times of crises warrants further investigation. However, from this study there are suggestions that acceptance of a degree of isolation by those in crises confers a positive means of coping together with the need to focus on reconnecting to social networks members once a crisis has passed. These findings imply that a greater understanding of the individuals' social network is required to avoid the unnecessary pathologizing of something that it likely to enhance recovery and provides greater understanding of the importance of social networks. This points to the need for services to understand and respect the resources that an individual already has in place in their social networks to help them cope with possible crises. Rather than agreeing with a traditional view that isolation is to be avoided, MH services might need to be aware that the personalized, use of isolation appears to be an essential part of crisis management and should be explored and supported rather than assumed to be a sign of illness. Acceptance of withdrawal, which can be monitored rather than services demanding social interaction at a time when it could be actively damaging, is worthy of consideration. In this respect, isolating behaviour is not necessarily a sign of further illness and may indicate a mechanism of healing and coping. Furthermore, the supportive, accepting and informative role that peers can play may be currently under‐utilized and estimated by services. Rather, the latter could provide and be considered an additional resource for supporting those in crisis and lends support to including peer workers who have lived experience of health problems to fulfil roles in MH care orientated to crises management.

### 4.1 Strengths and limitations

Whilst this study provides important insights into how people negotiate network members in crisis, there are some limitations. Recall of crisis for those not currently viewing themselves as in crisis may become distorted over time resulting in recall bias, and the small sample size limits the generalizability of the findings over larger groups of people. Individuals were predominantly recruited via a local recovery college and as such may not be representative of others in mental distress. All participants were white British and, where a preference was expressed, heterosexual. Thus, results cannot be generalized across minority groups based on ethnicity or sexuality. There is some evidence from other countries that some groups within LGBT communities and ethnic minorities are more at risk from MH crises and in need of supportive networks due to increased health needs coupled with limited access to social resources.^29^ Despite this, the study also has strengths. The in‐depth examination of the networks of people managing long‐term MH issues both day‐to‐day and in crisis provides a unique opportunity to further understand this area and to consider service crisis response in the light of the findings. It provides an enhanced view of the patient's world which could inform alternative and developing approaches to crisis management in MH services.

### 4.2 Conclusion and recommendations for research

A network perspective enables a view in which those with a MH problem at times of crises are seen as more than the subjects and objects of diminished social function. Social network membership and use appears to shrink from the broader diversity in everyday management, at times of crisis. The findings indicate the importance of exploring further the dynamics and interactions in networks as a means of understanding the responding to the support needs of people living with a MH problem. Future research might focus on the configuration and nature of personal networks for different groups in the population to understand how the navigation, negotiation and relational work undertaken in networks can promote or diminish access to resources at different times. The use of isolation as a personal crisis management tool needs further investigation as it may have implications for the management of MH. The findings of this study would appear to support the current move towards diversifying the current MH workforce, including the deployment of more peer and lay led ways of managing. Crisis support in MH services might fruitfully be reconsidered in the light of the finding that service use appears to be more helpful in the prevention of crisis and in helping people rebuild their lives after crisis.

## CONFLICT OF INTERESTS

The authors declare no conflict of interests.

## Note

1 Information on sexual preference was not gathered, but where participants disclosed a significant other, they were of the opposite gender.
